# MPK‐1/ERK is required for the full activity of resveratrol in extended lifespan and reproduction

**DOI:** 10.1111/acel.12867

**Published:** 2018-12-21

**Authors:** Dong Suk Yoon, Dong Seok Cha, Yoorim Choi, Jin Woo Lee, Myon‐Hee Lee

**Affiliations:** ^1^ Department of Medicine Brody School of Medicine at East Carolina University Greenville North Carolina; ^2^ Department of Orthopaedic Surgery Yonsei University College of Medicine Seoul South Korea; ^3^ Department of Oriental Pharmacy, College of Pharmacy Woosuk University Jeonbuk South Korea; ^4^ Brain Korea 21 PLUS Project for Medical Science Yonsei University College of Medicine Seoul South Korea; ^5^ Severance Biomedical Science Institute Yonsei University College of Medicine Seoul South Korea; ^6^ Lineberger Comprehensive Cancer Center University of North Carolina‐Chapel Hill Chapel Hill North Carolina

**Keywords:** ERK/MPK‐1, longevity, reproductive span, resveratrol, Sirtuin/SIR‐2.1, SKN‐1/NRF2

## Abstract

Resveratrol (RSV) extends the lifespan of various organisms through activation of sirtuin. However, whether RSV‐mediated longevity is entirely dependent upon sirtuin is still controversial. Thus, understanding additional mechanisms concerning the genetic requirements for the biological activity of RSV needs to be clarified to utilize the beneficial effects of RSV. In this study using *Caenorhabditis elegans* as a model system, we found that MPK‐1 (an ERK homolog) signaling is necessarily required for RSV‐mediated longevity of *sir‐2.1*/sirtuin mutants as well as for wild‐type worms. We demonstrated that MPK‐1 contributes to RSV‐mediated longevity through nuclear accumulation of SKN‐1 in a SIR‐2.1/DAF‐16 pathway‐independent manner. The positive effect of RSV in regulating lifespan was completely abolished by RNA interference against *mpk‐1* in the *sir‐2.1* and *daf‐16* mutants, strongly indicating that the MPK‐1/SKN‐1 pathway is involved in RSV‐mediated longevity, independently of SIR‐2.1/DAF‐16. We additionally found that RSV protected worms from oxidative stress via MPK‐1. In addition to organismal aging, RSV prevented the age‐associated loss of mitotic germ cells, brood size, and reproductive span through MPK‐1 in *C. elegans* germline. Therefore, our findings not only provide new mechanistic insight into the controversial effects of RSV on organismal longevity, but additionally have important implications in utilizing RSV to improve the outcome of aging‐related diseases.

## 1 INTRODUCTION

**Figure 1:**
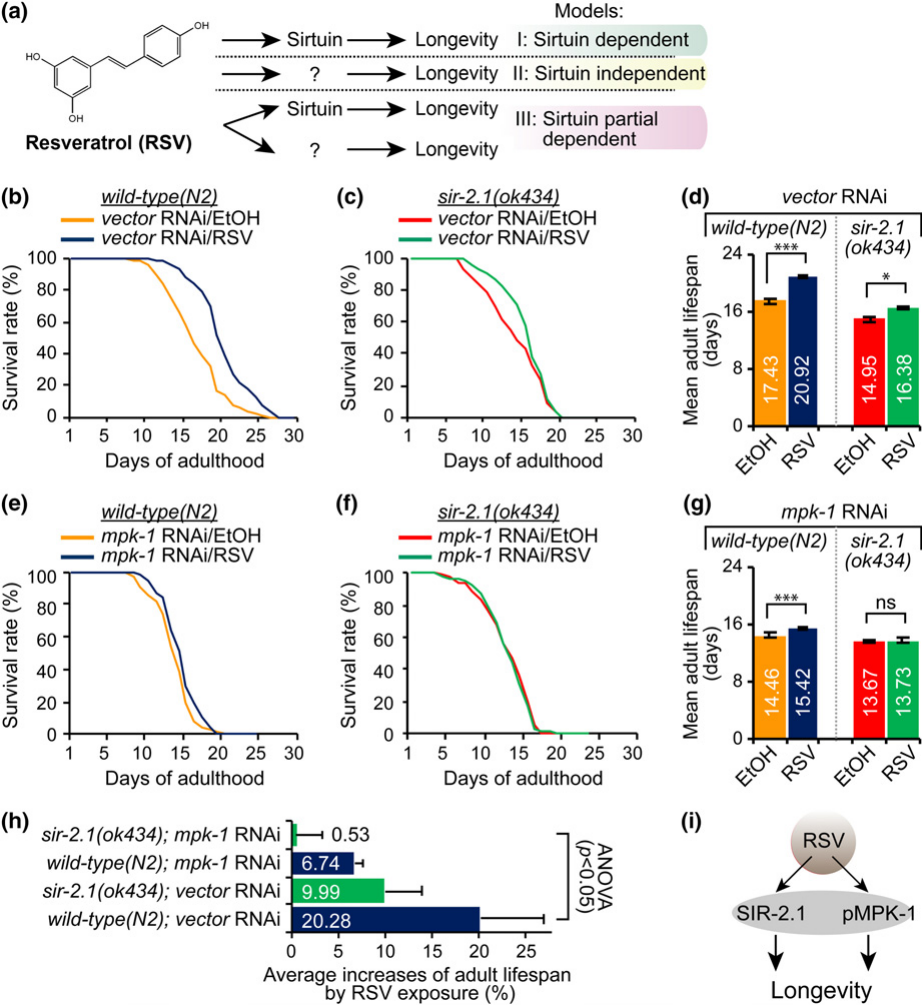
MPK‐1 is required for RSV‐mediated longevity of wild‐type (N2) and *sir‐2.1(ok434)* worms. (a) Potential action models for resveratrol (RSV)‐mediated longevity. (b−g) Adult lifespan curves and mean adult lifespan of wild‐type (b, d), *sir‐2.1(ok434)* (c, d), *mpk‐1 *RNAi in wild‐type (e, g), and *mpk‐1 *RNAi in *sir‐2.1(ok434) *null mutant worms (f, g) in the absence (0.1% EtOH as a vehicle) and presence of 100 μM RSV. (see Supporting Information Table S1; logrank test, *p < 0.05, **p < 0.01, and ***p < 0.001 RSV‐exposed worms compared with EtOH counterparts). (h) Average increases (%) of lifespan in worms exposed to RSV. ANOVA test (*p* < 0.05). See Supporting Information Table S3 for detailed statistical analysis. (i) This model shows that RSV exerts its longevity effect through two independent pathways, SIR‐2.1 and MPK‐1

Increasing evidence has shown that small molecules can affect the lifespan positively or negatively in a variety of organisms, including humans (Hubbard & Sinclair, 2014; Kennedy & Lamming, 2016). In particular, caloric restriction mimetics, including resveratrol (RSV), rapamycin, and metformin, have been shown to exert beneficial effects on longevity and health (Lamming, Sabatini, & Baur, 2012). Rapamycin was the first chemical identified which extends lifespan in mammals through the inhibition of mammalian target of rapamycin (mTOR; Harrison et al., 2009). Metformin, which can extend the lifespan of C57BL6 mice and short‐lived tumorigenic mice (Anisimov et al., 2005; Martin‐Montalvo et al., 2013), exerts its effects through inhibition of the mTOR signaling pathway (Dowling, Zakikhani, Fantus, Pollak, & Sonenberg, 2007). However, the mechanisms of RSV remain controversial even though its positive effects on longevity have been reported over the past decade**.** RSV was first identified as an activator of sirtuin (mammalian SIRT1/nematode SIR‐2.1, a family of NAD^+^‐dependent deacetylases; Figure 1a, *Model I*) and has been found to extend the lifespan of various organisms by mimicking dietary restriction and to improve the health of mice on a high‐fat diet (Baur et al., 2006; Wood et al., 2004). In addition, Herranz et al. (2010) reported the anti‐aging features of *Sirt1* by showing its suppressive effects on aging and metabolic disease using *Sirt1* transgenic mice. Thus, sirtuin activation has been thought to comprise an important mechanism of RSV‐mediated longevity. However, recent studies have highlighted the SIR‐2.1‐independent effects of RSV‐mediated longevity (Figure 1a, *Model II*). For example, (a) caloric restriction extends the lifespan independently of sirtuin in worms (Lee et al., 2006); (b) RSV has multiple targets including STAT3, JNK, AMPK, and ERK (Pirola & Frojdo, 2008). Among these, AMPK (5′‐AMP‐activated protein kinase) has been relatively well‐established as a target of RSV (Dasgupta & Milbrandt, 2007). RSV activates AMPK as its central target and acts indirectly on SIRT1 (Um et al., 2010). Therefore, sirtuin‐independent/indirect pathways or other RSV targets might be involved in RSV‐mediated longevity (Viswanathan, Kim, Berdichevsky, & Guarente, 2005); (c) SRT1720, known as a specific sirtuin activator which can ameliorate type 2 diabetes and metabolic diseases, can extend the lifespan and improve the health of mice (Mitchell et al., 2014). However, a contradictory effect of SRT1720 on longevity has additionally been reported, as SRT1720 cannot extend the lifespan and does not mimic the effect of RSV on lifespan extension in worms (Zarse et al., 2010). Therefore, the effect of RSV on lifespan extension may not function entirely in a sirtuin‐dependent manner. Moreover, it is necessary to identify other pathways or factors that respond to RSV (Figure 1a, *Model III*).

Resveratrol can bind to the integrin αVβ3 receptor to activate extracellular signal‐regulated kinases 1 and 2 (ERK1/2) in a human breast cancer cell line (Lin et al., 2006). In our previous study, using human mesenchymal stem cells, we observed that RSV increased the phosphorylation level of ERK, although this could vary depending on the number of cell passages (Yoon, Choi, Choi, Park, & Lee, 2015). It was reported that mitogen‐activated protein kinase‐1 (MPK‐1, known as human ERK homolog) extended the lifespan of *Caenorhabditis elegans* through SKN‐1 (the mammalian nuclear factor erythroid‐related factor; Okuyama et al., 2010). However, it has not yet been determined whether the RSV‐mediated lifespan extension in *C. elegans* can be regulated through MPK‐1 activity. In addition, to date, there have been no genetic studies clarifying the relationship between RSV and MPK‐1. Thus, the purpose of this study was to re‐evaluate the longevity effect of RSV‐mediated SIR‐2.1 and then to test whether MPK‐1/ERK, one of the candidate genes that respond to RSV, was involved with the longevity effect. Here, we demonstrate that RSV‐mediated longevity largely relies on two independent pathways, SIR‐2.1/DAF‐16 and MPK‐1/SKN‐1. Specifically, *mpk‐1* RNA interference (RNAi) completely abolished the longevity effect of RSV in *sir‐2.1* single null mutants. RSV exposure increased the level of phosphorylated MPK‐1 (pMPK‐1) and maintained the level of pMPK‐1 during aging in the wild‐type (WT) and *sir‐2.1* single null mutant nematodes. The RSV‐mediated MPK‐1 activation largely depended on the presence of SKN‐1 in a SIR‐2.1/DAF‐16‐independent manner. We additionally found that RSV‐mediated MPK‐1 activation increased reproductive span as well as delayed germline aging by maintaining mitotic germ cells.

## 2 RESULTS

### 2.1 ***mpk‐1* is required for RSV‐mediated longevity of *sir‐2.1* mutant as well as **WT **worms**

To re‐evaluate whether RSV‐mediated longevity depends entirely on SIR‐2.1, WT and *sir‐2.1(ok434)* null mutant worms on day 4 from embryos were cultured on nematode growth media (NGM) plates containing 100 μM RSV or 0.1% ethanol (EtOH) control at 20°C. The *sir‐2.1(ok434)* mutant worms were outcrossed four times prior to the main experiments (Supporting Information Figure S1a‐d). In agreement with a previous report (Bass, Weinkove, Houthoofd, Gems, & Partridge, 2007), RSV significantly extended the lifespan of WT worms (Figure 1b). RSV additionally extended the lifespan of *sir‐2.1(ok434)* mutants (Figure 1c). However, the RSV‐increased lifespan of *sir‐2.1(ok434)* mutants was less than that of WT worms (Figure 1d), suggesting that RSV‐mediated longevity is not entirely dependent upon *sir‐2.1*. A previous study demonstrated that the MPK‐1 pathway regulates longevity through SKN‐1 in *C. elegans *(Okuyama et al., 2010). To investigate whether MPK‐1 is involved in RSV‐mediated longevity, we examined the lifespan of WT worms in the absence or presence of RSV (vector or *mpk‐1* RNAi). Exposure to RSV led to an increase in the lifespan of *mpk‐1* RNAi‐treated WT worms (Figure 1e). However, the increased lifespan of *mpk‐1 *RNAi‐treated worms treated with RSV was likewise significantly less than that of WT worms (Figure 1e). This finding led us to test whether RSV‐mediated longevity may require both *sir‐2.1* and *mpk‐1*. We measured the lifespan of *sir‐2.1(ok434); mpk‐1 *(RNAi) worms. RSV failed to extend the lifespan of *sir‐2.1(ok434); mpk‐1 *(RNAi) worms (Figure 1f); no significant change in the mean lifespan (Figure 1g) and no increase in lifespan by RSV were observed (Figure 1h). We therefore concluded that both *sir‐2.1* and *mpk‐1* are critical for the full activity of RSV in regulating the lifespan of *C. elegans* (Figure 1i).

### 2.2 RSV maintains MPK‐1 activity throughout the lifespan of *C. elegans*

**Figure 2:**
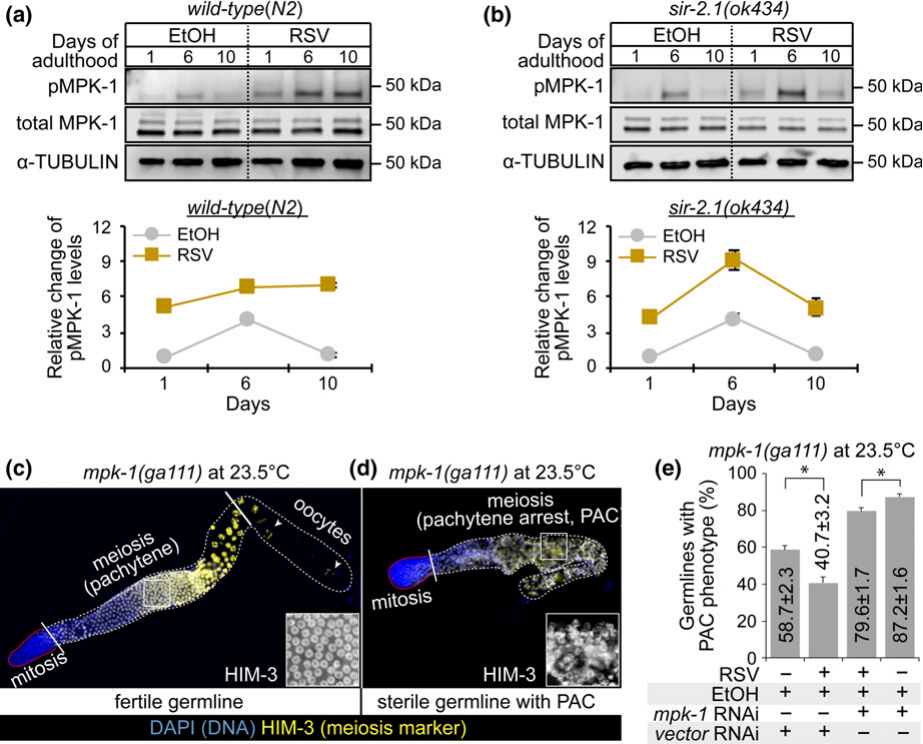
Resveratrol (RSV) activates MPK‐1 and rescues a sterile pachytene exit defect (PAC) phenotype in temperature‐sensitive *mpk‐1* mutants. Levels of total MPK‐1 and pMPK‐1 proteins were analyzed using extracts from wild‐type (a) and *sir‐2.1(ok434)* mutant worms (b) grown on NGM plates containing 100 μM RSV or vehicle (0.1% EtOH) at different time points (4, 10, and 14 days). α‐tubulin was used as a loading control. (c, d) Dissected germlines of adult hermaphrodites were stained with an anti‐HIM‐3 antibody (a marker for meiotic cells). Images were captured using consistent acquisition settings and under the same magnification. (c) Fertile *mpk‐1*(ts) germline. (d) Sterile *mpk‐1*(ts) germline with a PAC phenotype. The PAC germline exhibits abnormal pachytene cell morphology. (e) RSV rescued the PAC phenotype of *mpk‐1*(ts) mutants at 23.5°C. Gonads with the PAC phenotype were counted. The graph shows average percent of *mpk‐1*(*ts*) mutant worms with the PAC phenotype grown with EtOH‐ or RSV (100 μM)‐containing NGM agar plates under control or *mpk‐1 *RNAi. The percentage of control (RNAi)‐treated *mpk‐1*(ts) worms with the PAC phenotype in the absence and presence of RSV was 58.70 ± 2.25 (EtOH control, triplicate, *n* = 84) and 40.68 ± 3.16 (RSV, triplicate, *n* = 114), respectively (*p* = 0.028). The percentage of *mpk‐1*(RNAi)‐treated *mpk‐1*(ts) worms with the PAC phenotype in the absence and presence of RSV was 79.56 ± 1.70 (EtOH, triplicate, *n* = 69) and 87.19 ± 1.60 (RSV, triplicate, *n* = 62) (*p* = 0.028). *p*‐Values for each experimental group were calculated using the two‐tailed Student’s *t* test, and the error bars represent *SD*

We recently reported that RSV could activate ERK in human mesenchymal stem cells, depending on cell passage (Choi et al., 2018; Yoon et al., 2015). MPK‐1 has been shown to extend the lifespan of *C. elegans *(Okuyama et al., 2010). These findings led us to test whether MPK‐1 activity is regulated by RSV exposure. We confirmed that the levels of pMPK‐1 were significantly increased in WT worms exposed to 100 and 200 μM RSV (Supporting Information Figure S2). Next, WT and *sir‐2.1(ok434)* mutants were collected at three different time points. The results show that, upon RSV exposure, the levels of pMPK‐1 remained comparatively higher during aging in WT and *sir‐2.1(ok434)* mutant worms (Figure 2a,b). Next, to phenotypically reaffirm whether RSV induces MPK‐1 activation, we employed a temperature‐sensitive (*ts*) *mpk‐1(ga111)* loss‐of‐function mutant. The *mpk‐1(ts)* mutants are fertile at the permissive temperature (20°C) and have a sterile pachytene exit defect (PAC) phenotype that is caused by low levels of active MPK‐1 at the restrictive temperature (25°C; Leacock & Reinke, 2006). To test whether RSV rescues the PAC phenotype of *mpk‐1(ts) *by increasing MPK‐1 activity, L1‐stage *mpk‐1(ts)* mutants were grown on NGM agar plates containing RSV or EtOH control plates for four days at an intermediate temperature (23.5°C). The cellular morphology of meiotic germ cells was visualized by staining dissected gonads with HIM‐3 antibodies (a marker for meiotic cells; Figure 2c,d). A PAC phenotype was exhibited by 58.7% of *mpk‐1(ts)* mutant worms exposed to EtOH control, whereas 100 µM RSV exposure significantly reduced the percentage to 40.7% (Figure 2e). To confirm whether this reduction was a result of increased MPK‐1 protein activity, we utilized *mpk‐1 (RNAi)* in RSV‐ and EtOH‐treated *mpk‐1(ts)* mutant worms. The results show that *mpk‐1(RNAi)* inhibited RSV‐induced MPK‐1 activation and increased the percentage of worms with the PAC phenotype (Figure 2e). Thus, these findings strengthen the evidence that RSV promotes the activation of MPK‐1 in *C. elegans*.

### 2.3 MPK‐1 and SIR‐2.1 may have different downstream targets to promote RSV‐mediated longevity

**Figure 3:**
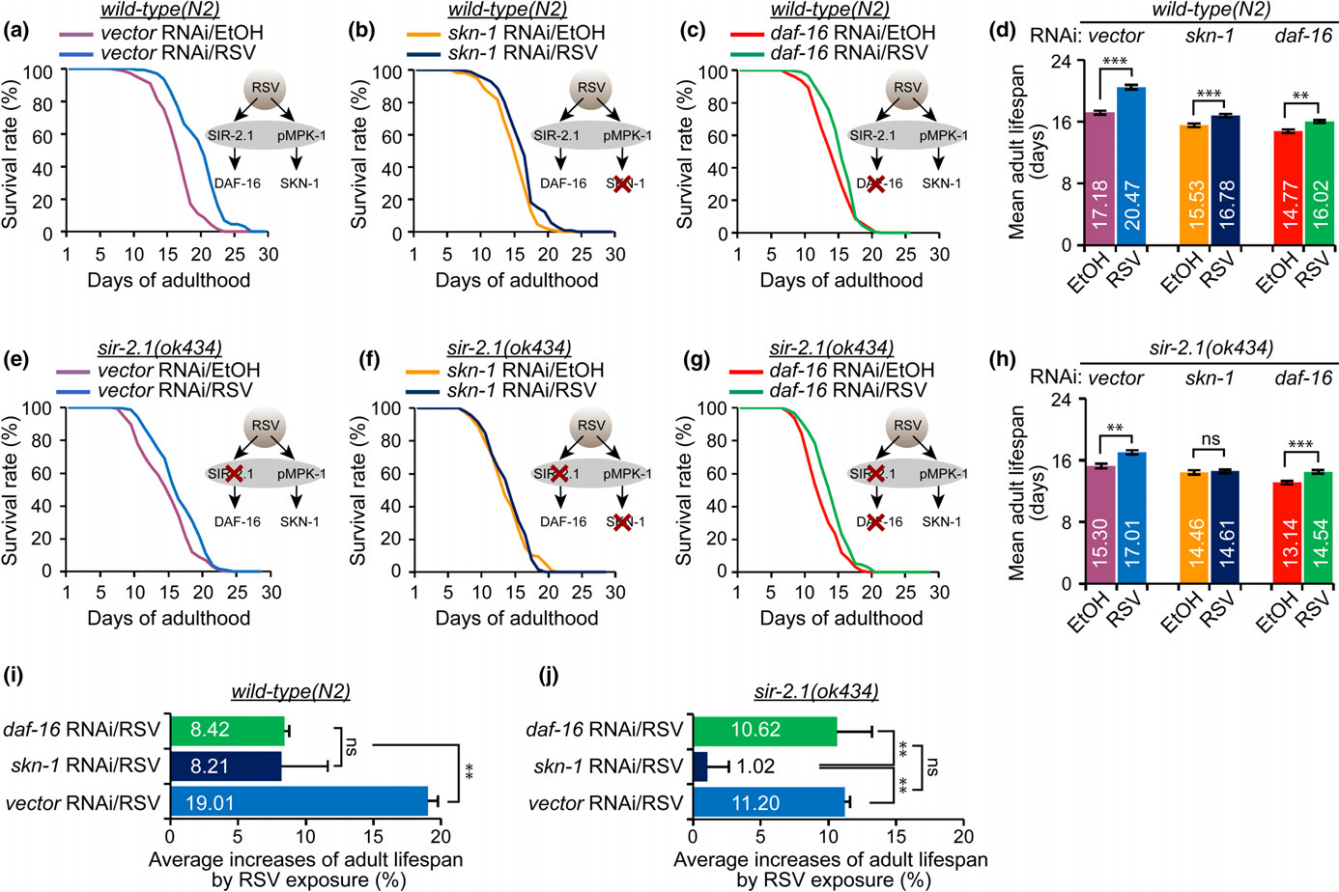
Knockdown of *skn‐1* completely abolishes the longevity effect of RSV in *sir‐2.1(ok434)*‐deficient worms. (a−c) Adult lifespan curves of wild‐type with RNAi vector control (a), wild‐type with *skn‐1* RNAi (b), and wild‐type with *daf‐16* RNAi (c) with vehicle (0.1% EtOH) and 100 μM resveratrol (RSV). (see Supporting Information Table S1). (d) Representative data are the means of three independent experiments (logrank test, *p < 0.05, **p < 0.01, and ***p < 0.001 RSV‐exposed worms compared to EtOH counterparts). (e−g) Adult lifespan curves of *sir‐2.1(ok434)* with RNAi vector control (e), *sir‐2.1(ok434)* with *skn‐1* RNAi (f), and *sir‐2.1(ok434)* with *daf‐16* RNAi (g) with vehicle (0.1% EtOH) and 100 μM RSV. (see Supporting Information Table S1). (h) Representative data are the means of three independent experiments (logrank test, *p < 0.05, **p < 0.01, and ***p < 0.001 RSV‐exposed worms compared to EtOH counterparts). (i, j) Average increases (%) of adult lifespan in worms exposed to RSV. ANOVA test (*p* < 0.05). See Supporting Information Table S3 for detailed statistical analysis

It has been reported that active MPK‐1 phosphorylates key residues of SKN‐1 protein, which is required for normal lifespan in *C. elegans *(An & Blackwell, 2003). Furthermore, MPK‐1/SKN‐1‐mediated longevity in *C. elegans* is shown to be independent of DAF‐16, which is known as a downstream regulator of SIR‐2.1 in regulating lifespan (Berdichevsky, Viswanathan, Horvitz, & Guarente, 2006; Tissenbaum & Guarente, 2001; Wang et al., 2006). Thus, we wanted to identify whether SKN‐1 and DAF‐16 are involved together or separately in RSV‐mediated longevity. To evaluate this, we employed WT and *sir‐2.1*(*ok434*) mutant worms for RNAi experiments involving *skn‐1* and *daf‐16*. RSV led to an increased lifespan in each *skn‐1* or *daf‐16 *knockdown worms (Figure 3a−c). However, the increased lifespan of *skn‐1* or *daf‐16* RNAi‐treated worms exposed to RSV was significantly less than that of WT worms (Figure 3d,i). Thus, this result indicates that both SKN‐1 and DAF‐16 are partially involved in RSV‐mediated longevity. Next, we measured the lifespan of *sir‐2.1*(*ok434*); *skn‐1(RNAi)* and *sir‐2.1*(*ok434*); *daf‐16(RNAi)* worms. RSV failed to extend the lifespan of *sir‐2.1(ok434); skn‐1(RNAi)* worms (Figure 3e,f); no significant change in the mean lifespan (Figure 3h) and no increase in lifespan by RSV were observed (Figure 3j). In contrast, RSV increased the lifespan of *sir‐2.1(ok434); daf‐16(RNAi)* worms to a level similar to that of *sir‐2.1(ok434); control vector (RNAi)* (Figure 3e,g,h,j). At this point, we concluded that SKN‐1 is required for RSV‐mediated longevity in a SIR‐2.1‐independent manner, whereas DAF‐16 has been shown to be involved in the RSV‐mediated SIR‐2.1 activation.

### 2.4 MPK‐1 contributes to RSV‐mediated longevity via SKN‐1, independently of SIR‐2.1/DAF‐16 pathway

**Figure 4:**
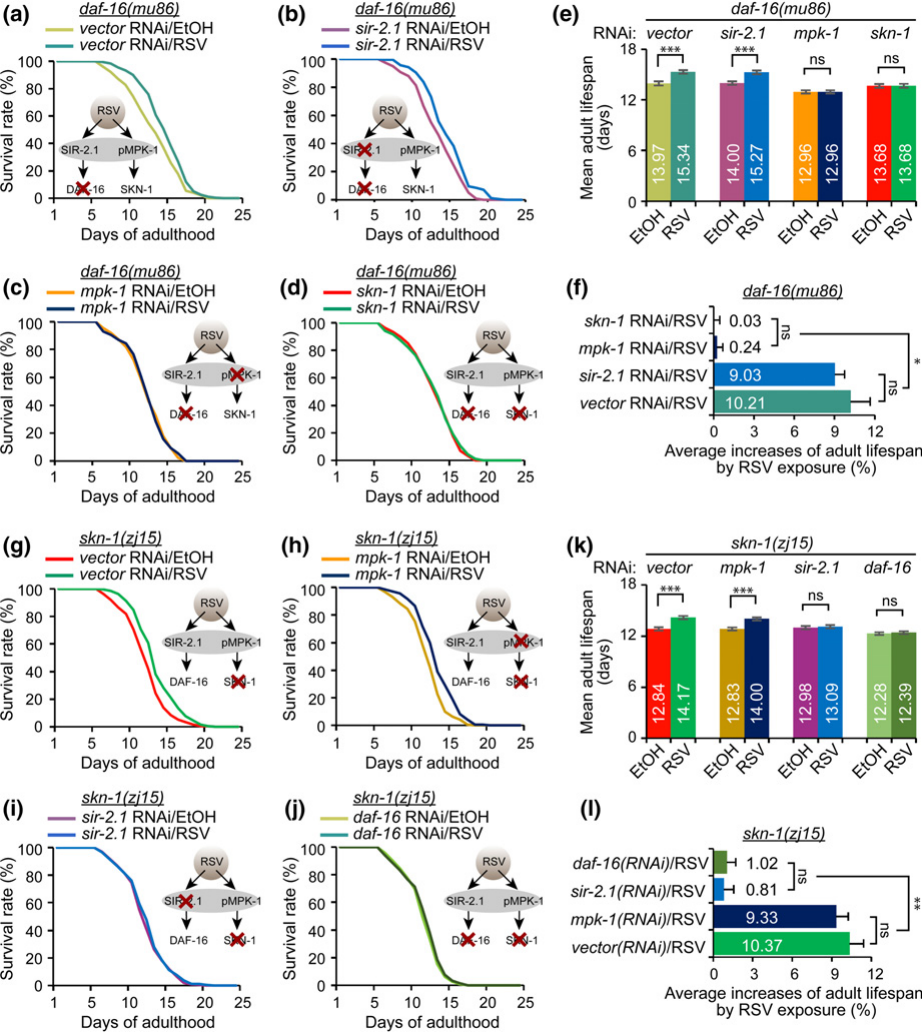
Resveratrol (RSV)‐activated MPK‐1 contributes to longevity in a SKN‐1‐dependent manner, but not in SIR‐2.1 and DAF‐16‐dependent fashions. Adult lifespan curves of *daf‐16(mu86)* with RNAi vector control (a), *daf‐16(mu86)* with *sir‐2.1* RNAi (b), *daf‐16(mu86)* with *mpk‐1* RNAi (c), and *daf‐16(mu86)* with *skn‐1* RNAi (d) with vehicle (0.1% EtOH) and 100 μM RSV. (see Supporting Information Table S1). (e) The representative data are the means of three independent experiments (logrank test, *p < 0.05, **p < 0.01, and ***p < 0.001 RSV‐exposed worms compared with EtOH counterparts). (f) Average increases (%) of adult lifespan in the worms exposed to RSV. ANOVA test (*p* < 0.05). See Supporting Information Table S3 for detailed statistical analysis. Adult lifespan curves of *skn‐1(zj15)* with RNAi vector control (g), *skn‐1(zj15)* with *mpk‐1* RNAi (**h**), *skn‐1(zj15)* with *sir‐2.1* RNAi (i), and *skn‐1(zj15)* with *daf‐16* RNAi (j) in the absence (0.1% EtOH as a vehicle) and presence of 100 μM RSV (see Supporting Information Table S1). (k) The representative data are the means of three independent experiments (logrank test, *p < 0.05, **p < 0.01, and ***p < 0.001 RSV‐exposed worms compared with EtOH counterparts). (l) Average increases (%) of adult lifespan in the worms exposed to RSV. ANOVA test (*p* < 0.05)

DAF‐16 can be activated by SIR‐2.1 to extend *C. elegans* lifespan (Berdichevsky et al., 2006; Mouchiroud et al., 2013), whereas RSV‐mediated MPK‐1 extends the lifespan of *C. elegans* through SKN‐1 regulation (Okuyama et al., 2010; Figures 1f and 3f). However, whether MPK‐1 and SIR‐2.1 have independent downstream targets under RSV stimulation has not been studied. To examine the possible mechanisms of MPK‐1 in RSV‐mediated lifespan extension, *daf‐16*(*mu86*) and *skn‐1*(*zj15*) mutant worms were employed. RSV led to a small increase in lifespan extension of *daf‐16(mu86)* mutant worms (Figure 4a,e,f). RNAi‐mediated knockdown of *sir‐2.1* did not affect the increase in the lifespan effected by RSV in *daf‐16(mu86)* worms (Figure 4b,e,f). However, knockdown of *mpk‐1* or *skn‐1* completely blocked RSV‐induced lifespan extension in *daf‐16(mu86)* worms (Figure 4c‐f). Thus, we conclude that MPK‐1 and SKN‐1 influence RSV‐mediated longevity by acting independently of DAF‐16, whereas SIR‐2.1 has been shown to be dependent on the presence of DAF‐16 under RSV stimulation. RSV additionally extended the lifespan of *skn‐1(zj15)* mutant worms (Figure 4g,k,l). As expected, knockdown of *mpk‐1* did not affect the RSV‐mediated increase in the average lifespan (Figure 4h,k,l), whereas knockdown of *sir‐2.1* or *daf‐16* completely blocked the RSV‐mediated lifespan extension in *skn‐1(zj15)* mutant worms (Figure 4i‐l), thereby strongly indicating that the MPK‐1‐mediated longevity effect is dependent upon the presence of *skn‐1* under RSV stimulation, but independent of the presence of *sir‐2.1* and *daf‐16*. Reactive oxygen species (ROS) generate byproducts of normal oxidative metabolism which cause an accumulation of molecular damage, resulting in acceleration of aging processes (Horne & Ricciardo, 1988). It is well‐known that SIR‐2.1 binds nuclear DAF‐16 to promote DAF‐16‐dependent transcription, stress resistance, and longevity (Berdichevsky et al., 2006). Although Okuyama et al. (2010) found that MPK‐1 phosphorylates the key site of SKN‐1 required for nuclear accumulation and SIR‐2.1, DAF‐16, and SKN‐1 have been implicated in the ROS‐mediated aging processes (Berdichevsky et al., 2006; Blackwell, Steinbaugh, Hourihan, Ewald, & Isik, 2015; Liu et al., 2015; Ye et al., 2010), whether MPK‐1 is involved in regulating oxidative stress under RSV stimulation remains undetermined. To clarify whether MPK‐1 activation is involved in the RSV‐mediated protection of worms from oxidative stress, a time‐course experiment was performed to measure the level of intracellular ROS using the molecular probe H2DCF‐DA with a 32°C heat shock. The results revealed that RSV attenuated ROS accumulation in WT worms and that silencing of *sir‐2.1* partially abolished the RSV effect in preventing ROS accumulation from heat shock (Supporting Information Figure S3a,b). Knockdown of *mpk‐1 *additionally partially weakened the RSV effect in WT worms, whereas in *sir‐2.1(ok434)* mutants, the effect was completely abolished by *mpk‐1 *knockdown (Supporting Information Figure S3c,d). These data show that RSV‐activated MPK‐1 plays a role in RSV‐enhanced oxidative stress resistance. Thus, we suggest that the MPK‐1/SKN‐1 pathway is activated in a SIR‐2.1‐independent manner under RSV stimulation, but the role may be identical, at least in this part, for protecting the worms from ROS accumulation.

### 2.5 MPK‐1 is involved in the RSV‐mediated nuclear accumulation of SKN‐1 independently of SIR‐2.1

**Figure 5:**
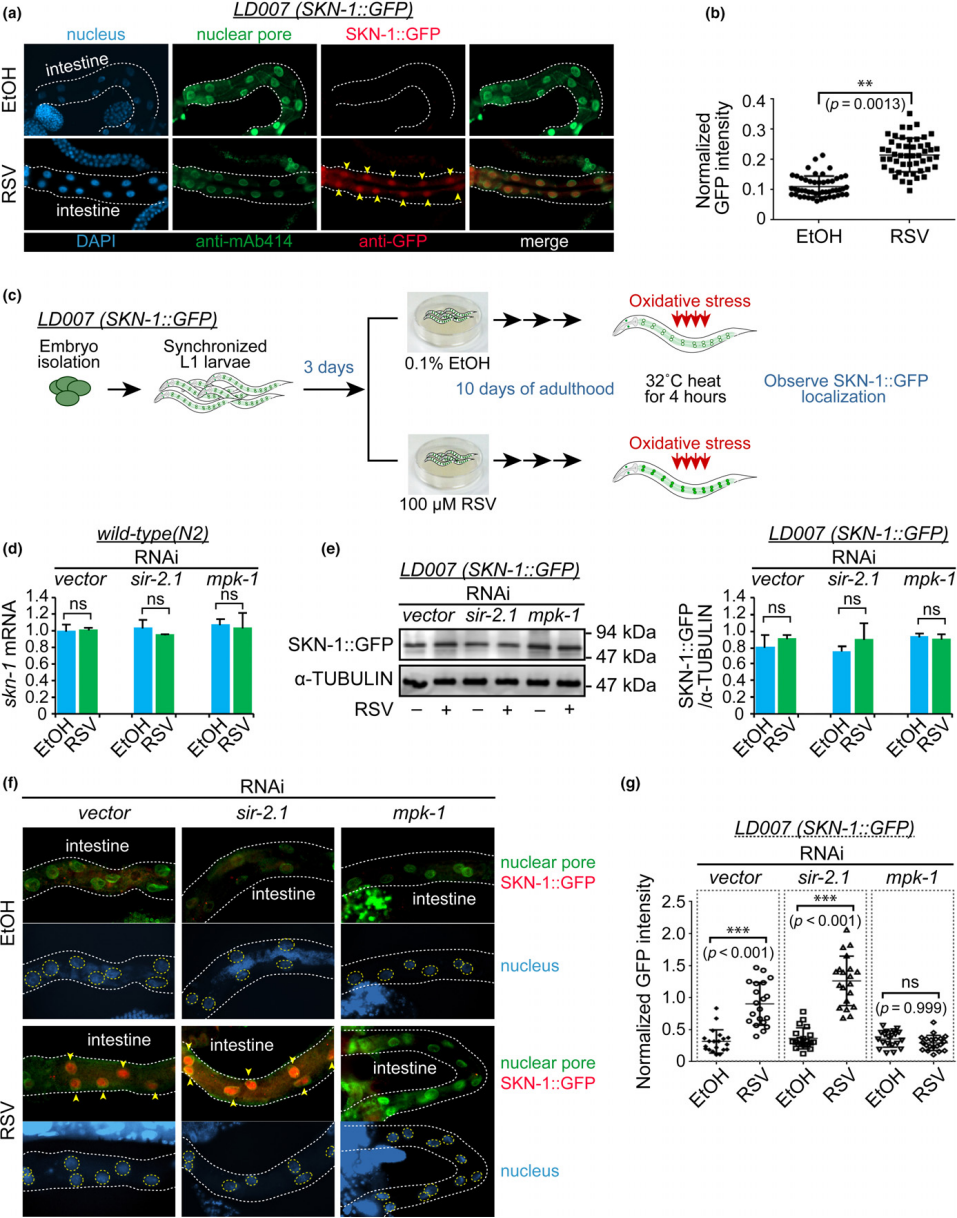
Nuclear accumulation of SKN‐1 by resveratrol (RSV) depends on the presence of *mpk‐1*. (a) SKN‐1(b/c)::GFP transgenic worms treated with 0.1% EtOH or 100 µM RSV were dissected, and the worms were stained with antibodies against GFP or nuclear pore complex proteins (Mab414). All images were acquired using consistent settings and under the same magnification. (b) Quantitative analysis of the GFP‐positive nuclei was performed using ImageJ. **p < 0.01 compared with EtOH‐treated SKN‐1::GFP transgenic worms. The *p*‐values were calculated using the two‐tailed unpaired Student's *t* test, and the error bars indicate *SD* *n* = 50 for each group. (c) Embryos in SKN‐1(b/c)::GFP transgenic worms were isolated and then maintained for 4 days on OP50 NGM plates. On day 4 after embryo isolation, the worms were transferred to each RNAi pate containing 0.1% EtOH or 100 µM RSV and then maintained for an additional 10 days. At day 10 of adulthood, the worms were placed in an incubator at 32°C for 4 hr, then collected for western blotting, and dissected and fixed for immunostaining. (d) mRNA expression of *skn‐1* was determined in wild‐type (N2) worms on day 10 of adulthood. Likewise, on day 4 from embryo isolation, the worms were transferred to each RNAi pate containing 0.1% EtOH or 100 µM RSV and then maintained for an additional 10 days. The worms were placed in an incubator at 32°C for 4 hr and then collected for qRT–PCR. The qRT–PCR assay was performed in triplicate (*ns* denotes no significance). (e) Protein level of SKN‐1 was determined by western blot assay in triplicate, and the quantification was performed using the ImageJ program (*ns*, no significance). (f) The dissected and fixed worms were stained with antibodies against GFP or nuclear pore complex proteins (Mab414). All images were acquired using consistent settings under the same magnification. (g) Quantitative analysis of the GFP‐positive nuclei was performed using ImageJ. **p < 0.01 compared with EtOH‐treated SKN‐1::GFP transgenic worms. The *p*‐values were calculated using the one‐way ANOVA test, and the error bars indicate *SD* (*n* = 20 for each group)

It is well‐known that SIR‐2.1 binds nuclear DAF‐16 to promote DAF‐16‐dependent transcription, stress resistance, and longevity (Berdichevsky et al., 2006). Although Okuyama *et al.* found that MPK‐1 phosphorylates the key site of SKN‐1 required for nuclear accumulation (Okuyama et al., 2010), it has not been determined yet whether MPK‐1 induces nuclear import of SKN‐1. To clarify whether MPK‐1 regulates cellular localization of SKN‐1 under RSV stimulation, we employed a SKN‐1::GFP [*ldIs7;skn‐1b/c::GFP+rol‐6(su1006)*] strain. First, we checked whether SKN‐1::GFP localization was changed under RSV stimulation through immunostaining. RSV significantly induced the nuclear accumulation of SKN‐1 in the intestine (Figure 5a,b). We then examined whether *sir‐2.1* and *mpk‐1* are involved in the RSV‐induced accumulation of nuclear SKN‐1. To test this, worms were synchronized at L1 stage and maintained under normal growth conditions until they reached adults (at 4 days after embryo stage), and then the adult worms were transferred to EtOH‐ or RSV‐containing RNAi plates. The transferred worms were kept on the EtOH‐ or RSV‐containing RNAi plates for 10 days and then subjected to heat stress for 4 hr at 32°C (Figure 5c). We first checked the mRNA and protein levels of *skn‐1* using real‐time qPCR and western blot analysis. Neither RSV nor RNAi of *mpk‐1* or *sir‐2.1* affected the mRNA and protein levels of *skn‐1* (Figure 5d,e). Knockdown of *sir‐2.1* by RNAi did not affect the nuclear accumulation of SKN‐1 induced by RSV (Figure 5f), which indicates that the nuclear import of SKN‐1 does not depend on the presence of *sir‐2.1*. However, RNAi‐mediated knockdown of *mpk‐1* completely blocked RSV‐induced nuclear accumulation of SKN‐1::GFP (Figure 5f). Quantitative analysis of SKN‐1::GFP translocation following RSV stimulation in *mpk‐1* RNAi‐treated worms showed no difference between EtOH‐ and RSV‐treated groups (Figure 5g). To investigate whether RSV‐induced MPK‐1 is related to the presence of DAF‐16, we examined the mRNA and protein levels of DAF‐16. Neither RSV nor RNAi knockdown of *mpk‐1* or *sir‐2.1* affected the mRNA expression of *daf‐16* (Supporting Information Figure S4a). Interestingly, RSV increased the protein level of DAF‐16 in WT worms treated with vector or *mpk‐1* RNAi, whereas *sir‐2.1* inhibition by RNAi completely blocked the RSV‐mediated increase in DAF‐16 protein (Supporting Information Figure S4b,c). In addition, RSV treatment increased the mRNA levels of *sod‐3* and *hsp‐16.2*, which are well‐known downstream targets of DAF‐16 (Wang, Zhang, Lu, & Zhou, 2015). As expected, RSV increased the expression of the DAF‐16 target mRNAs in the worms treated with vector control or *mpk‐1* RNAi, which was blocked by knockdown of *sir‐2.1* by RNAi (Supporting Information Figure S4d). These results suggest that RSV‐mediated MPK‐1 action is independent of the SIR‐2.1/DAF‐16 pathway. However, we demonstrated that the RSV‐induced accumulation of SKN‐1 is dependent on *mpk‐1*.

### 2.6 RSV prevents germline aging via MPK‐1

**Figure 6:**
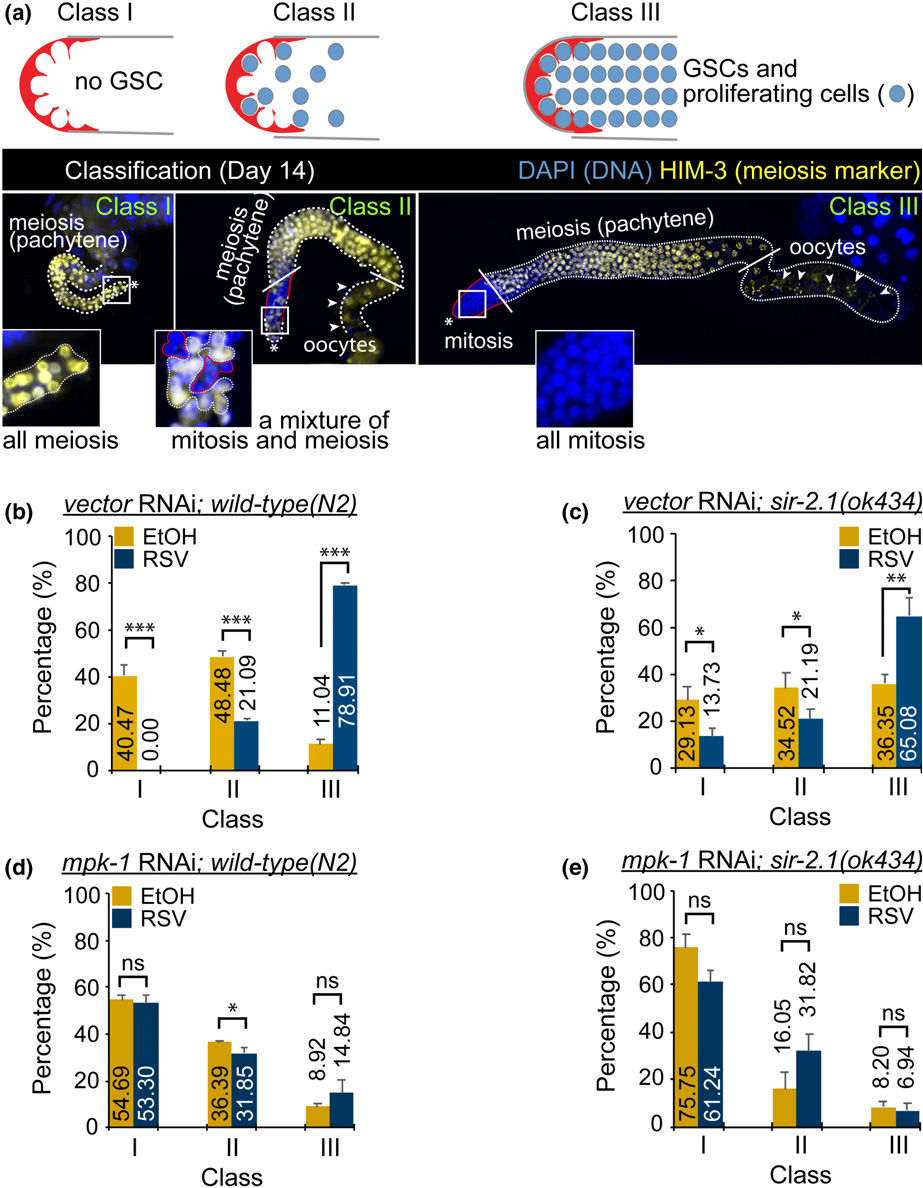
Effects of resveratrol (RSV) on *Caenorhabditis elegans* germline aging via MPK‐1. (a) Dissected germlines of 14‐day‐old adult hermaphrodites were stained with an antibody against HIM‐3. All images were acquired using consistent settings under the same magnification. A picture in the class I panel shows a smaller gonad and all HIM‐3‐negative/meiotic cells (see the magnified inset of the picture in Figure 6a). An image on the class II panel illustrates a middle‐sized gonad, in which oocytes are occasionally observed. The class II gonads possess a mixture of mitotically dividing cells (HIM‐3‐negative) and meiotic cells (HIM‐3‐positive) in the most distal germline (see the magnified image of the inset in Figure 5b; dashed white lines indicate HIM‐3‐positive cells, and red lines indicate HIM‐3‐negative cells). A picture in the class III panel shows gonads larger in size compared with the other two groups, class I and II. All of the class III gonads possess oocytes and HIM‐3 negative mitotic germ cells in the distal region of the germline. *, distal end; solid red lines, mitotic cells (HIM‐3‐negative); dashed white lines, meiotic cells (HIM‐3‐positive); solid white lines, a boundary between mitosis and meiosis as well as a boundary between meiotic pachytene and maturing oocytes region; arrowheads, individual oocytes. Percentages of each germline classified as class I, II, or III in wild‐type with RNAi vector control (b), *sir‐2.1(ok434)* with RNAi vector control (c), wild‐type with *mpk‐1* RNAi (d), and *sir‐2.1(ok434)* with *mpk‐1* RNAi (e) worms that were exposed to vehicle (0.1% EtOH) or to RSV during the 14 days after the embryonic stage. All scores shown in the graphs are the means of three independent experiments. *p‐*Values for each experimental group were calculated using the two‐tailed Student's *t* test, and the error bars indicate *SD*

Germline stem cell (GSC) capacity is diminished, and germ cell numbers are decreased during the aging process (Qin & Hubbard, 2015). Thus, we wanted to investigate the potential effect of RSV on the maintenance of GSC capacity during aging. For more detailed analysis, germline phenotypes were classified into three groups depending on (I) germline size, (II) GSC proliferation capacity, and (III) the presence of gametes (e.g., oocytes; see Figure 6a legend for more details). We found that WT worms exposed to RSV contained more class III germlines and significantly reduced percentages of class I and II germlines than those of worms treated with EtOH and at day 10 of the adult stage (Figure 6b). We next investigated the effect of RSV on *sir‐2.1(ok434)* germline aging. The results show that *sir‐2.1(ok434)* mutant worms exposed to RSV likewise had more class III and slightly reduced percentages of class I and II germlines compared to those of the EtOH‐treated worms at day 14 (Figure 6c). We then examined whether MPK‐1 is required for this RSV‐mediated effect delaying germline aging. RNAi‐mediated knockdown of *mpk‐1* in WT and *sir‐2.1(ok434)* worms clearly blocked the effects of RSV, which can delay germline aging. There were no differences between EtOH‐ and RSV‐treated groups, especially in class III gonads (Figure 6d,e). This result suggests that the positive effects of RSV in maintaining mitotic GSCs depend entirely on the presence of MPK‐1 throughout the worm’s lifespan.

### 2.7 *mpk‐1* is required for RSV‐mediated reproductive longevity

**Figure 7:**
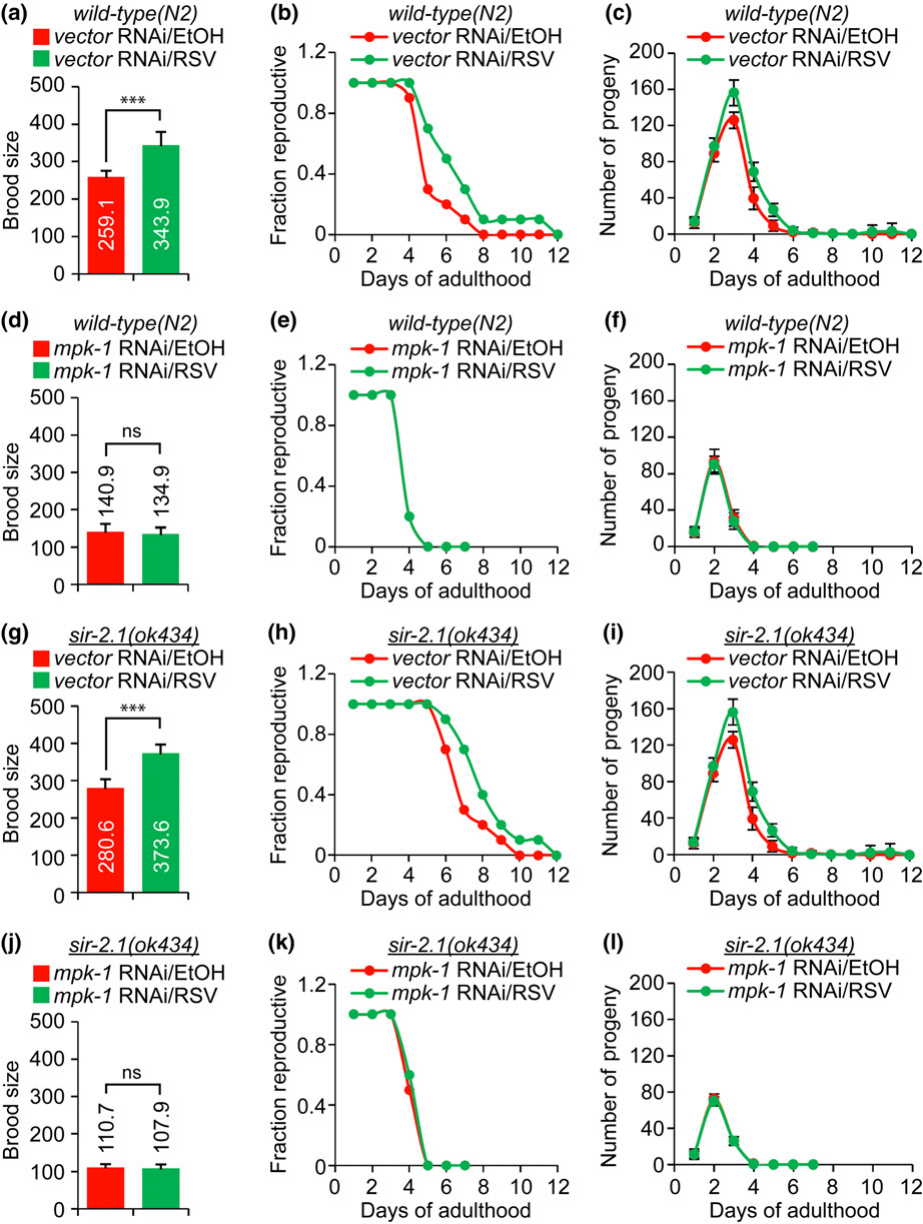
Physiological effects of resveratrol (RSV) on the reproductive system of *Caenorhabditis elegans*. (a) Brood size, (b) reproductive span curves, and (c) daily progeny production of wild‐type (N2) worms in the absence (0.1% EtOH as a vehicle) and presence of 100 μM RSV. (d) Brood size, (e) reproductive span curves (there is no difference between EtOH and RSV groups; hence, it only shows the *mpk‐1* RNAi/RSV response; the EtOH and RSV curves are superimposed over each other), and (f) daily progeny production of the *mpk‐1* RNAi‐treated wild‐type worms in the absence (0.1% EtOH as a vehicle) and presence of RSV. (g) Brood size, (h) reproductive span curves, and (i) daily progeny production of *sir‐2.1* (*ok434*) mutant worms in the absence (0.1% EtOH as a vehicle) and presence of RSV. (j) Brood size, (k) reproductive span curves, and (l) daily progeny production of the *mpk‐*1 RNAi‐treated sir*‐2.1* (*ok434*) mutant worms in the absence (0.1% EtOH as a vehicle) and presence of RSV. (a, d, g, e) The representative data are the means of three independent experiments (Student's *t* test, ***p < 0.001; RSV‐exposed worms compared with EtOH counterparts, *ns*; not significant). (b, e, h, k) The representative data are the means of three independent experiments (ANOVA test, b, h; *p* < 0.01, e, k; *ns*). (c, f, i, l) The representative data are the means of three independent experiments (ANOVA test, c, i; *p* < 0.05, f, l; *ns*)

As mentioned above, RSV maintained healthy germ cells during aging. To clarify the physiological significance of this RSV effect in the maintenance of mitotic germ cells during aging, we examined the effects of RSV on progeny production on aging. WT and *sir‐2.1(ok434)* mutant worms were synchronized at the L1 stage by embryo isolation and then grown on until they reached the L4 stage. The worms were then transferred to a new NGM plate with a single worm per plate. The next day, worms capable of producing progeny were transferred to EtOH‐ or RSV‐containing plates in seeded with RNAi bacteria. Worms that died during the reproductive periods were excluded in this experiment. The worms were transferred to new plates daily and viable progenies on the plates were counted 2 days after the worms were moved to a new plate. The results show that RSV clearly increased the brood size and reproductive span of WT worms (Figure 7a,b). The number of viable progeny was likewise increased by RSV treatment during the reproductive period (Figure 7c). As expected, *mpk‐1* inhibition by RNAi abolished the effects of RSV as well as decreased brood size, reproductive span, and number of viable progeny (Figure 7d−f). Similarly, RSV increased brood size, reproductive span, and number of viable progeny in the *sir‐2.1(ok434)* mutant worms (Figure 7g−i), but RNAi of *mpk‐1* blocked this effect (Figure 7j−l). Collectively, these results indicate that MPK‐1 contributes to the prolonged reproductive span induced by RSV through maintenance of mitotic germ cells during reproductive aging. Thus, we suggest that the RSV/MPK‐1 pathway might be a new target for germline health.

## 3 DISCUSSION

Resveratrol was originally identified as an activator of sirtuin and its invertebrate homologs, and has been shown to extend lifespan in both invertebrates and vertebrates (Bhullar & Hubbard, 2015; Pallauf, Rimbach, Rupp, Chin, & Wolf, 2016). However, subsequent studies have suggested that RSV‐mediated longevity may be independent of sirtuin and may not stimulate sirtuin activity to promote longevity (Hu, Liu, Wang, & Liu, 2011). Therefore, the effects of RSV on longevity seem to be controversial. In this study, we provide important new insights into the effects of RSV on longevity and GSC aging using *C. elegans*. Previous findings have shown that RSV can extend the lifespan of yeast, worms, and mice in a sirtuin‐dependent manner (Wood et al., 2004). This positive effect of RSV‐mediated sirtuin activation on lifespan extension has been explored in various research fields, such as stem cell aging, diabetes, and cancer (Buhrmann, Shayan, Popper, Goel, & Shakibaei, 2016; Cote et al., 2015; Liu et al., 2012). For these reasons, the effects of RSV‐mediated sirtuin activity cannot be overlooked, although some studies reported that the role of RSV as a sirtuin activator still remains controversial and other alternative pathways need to be found and characterized. Viswanathan et al. (2005) reported that the RSV effect on *C. elegans* longevity is completely dependent upon *sir‐2.1*, but independent of *daf‐16*. Nevertheless, they suggested that RSV inhibits SIR‐2.1 activity to prevent *sir‐2.1*‐mediated *abu‐11* repression. Other studies have shown that SRT1720, a sirtuin‐specific activator, did not extend the lifespan of *C. elegans *(Zarse et al., 2010). In this study, we observed that RSV partially induced the lifespan extension of *C. elegans* by *sir‐2.1(ok434)* mutation. RSV has multiple putative targets, including STAT3, JNK, AMPK, and ERK, among others (Pirola & Frojdo, 2008). Of these, AMPK has been relatively well‐established as an RSV target (Dasgupta & Milbrandt, 2007). One study showed that RSV activates AMPK as its central target and acts indirectly on SIRT1. Additionally, RSV‐mediated AMPK activation is dependent on SIRT1 (Um et al., 2010). Furthermore, RSV cannot induce SIRT1 activation in the absence of AMPK. Hence, it is thought that AMPK is a mediator of RSV‐induced sirtuin activation. In contrast, Dasgupta and Milbrandt (2007) showed that neuronal activation of AMPK by RSV does not require the presence of SIRT1. Thus, it is controversial whether RSV‐mediated AMPK action is dependent upon the presence of SIRT1. In *C. elegans*, lifespan can be regulated by *aak‐2*, a gene encoding the AMPK protein (Apfeld, O'Connor, McDonagh, DiStefano, & Curtis, 2004). SIR‐2.1 extends the lifespan of *C. elegans* via both *aak‐2*‐dependent and *aak‐2*‐independent mechanisms (Curtis, O'Connor, & DiStefano, 2006). DAF‐16 is known to be a longevity factor in *C. elegans *(Lin, Hsin, Libina, & Kenyon, 2001). However, it was reported that RSV‐mediated lifespan extension was not dependent on DAF‐16 (Viswanathan et al., 2005). Thus, it is possible that RSV functions through an additional mechanism to extend the lifespan of *C. elegans* in a SIR‐2.1‐independent manner. Our results show that *mpk‐1* is required for the RSV‐mediated lifespan extension in *C. elegans*. Knockdown of *mpk‐1* in *sir‐2.1(ok434)* mutant worms completely abolished RSV‐mediated lifespan extension, indicating that MPK‐1 operates independently of SIR‐2.1 in RSV‐mediated lifespan extension. MPK‐1 was first identified as a longevity factor in *C. elegans* by Okuyama et al. (2010). They reported that active MPK‐1 phosphorylates key residues required for nuclear import of SKN‐1, which is required for normal lifespan (An & Blackwell, 2003). They additionally showed that MPK‐1 extended the lifespan of *C. elegans* independently of *bar‐1 *(β‐catenin human homolog), *hsf‐1 *(Heat shock protein human homolog), and *sir‐2.1*, which are known as regulators related to *daf‐16*‐dependent lifespan regulation (Essers et al., 2005; Hsu, Murphy, & Kenyon, 2003). Our lifespan results support this mechanism in that double mutation of *sir‐2.1* and *mpk‐1* completely abolished the RSV‐mediated lifespan extension, thereby indicating that *mpk‐1* contributes to the RSV‐mediated lifespan extension independently of *sir‐2.1*. Our findings suggest that both *sir‐2.1* and *mpk‐1* are required for RSV‐mediated lifespan extension and that MPK‐1 is a longevity determinant which acts independently of SIR‐2.1 in *C. elegans*.

In addition to somatic aging, several studies have shown that the functioning of the reproductive system declines with age. During developmental processes in *C. elegans*, mitotic germ stem cells, termed GSCs, or progenitors, are located farthest from distal end. The cells enter meiosis and then are differentiated into sperm (L4 stage) or oocytes (adulthood stage). The number of these mitotic germ cells decreases with age (Qin & Hubbard, 2015). However, the biological mechanisms underlying this process remain poorly understood. Moreover, the effect of RSV on age‐associated stem cell loss has not yet been studied in other systems. Our current study demonstrates that RSV can delay germline aging by maintaining mitotic cells at distal regions and maintaining proliferative capacity (Figure 6). We did not observe mitotic germ cells (HIM‐3‐negative cells) in most WT or *sir‐2.1(ok434)* mutant worms by day 10 of adult stage. Therefore, it seems that mitotic germ cells of *C. elegans* gonads lose their self‐renewal potential with age. In addition, oocytes were not observed in the aged gonads, indicating that meiotic cells lose their potential to differentiate into the oocyte lineage. Most stem cells lose their self‐renewal and differentiation potentials with age (Oh, Lee, & Wagers, 2014). Thus, it is thought that the *C. elegans* germline is a good in vivo model system for study of stem cell aging (Hubbard, 2007). Our current study shows that RSV can prolong the in vivo maintenance of germline mitotic cells of *C. elegans* during aging, and this positive effect of RSV on germline mitotic cell maintenance is lost in *mpk‐1 *knockdown worms (Figure 6d,e). MPK‐1 has multiple functions in the *C. elegans* germline, such as germ cell fate specification and membrane organization of pachytene cells. Most processes during germline development of *C. elegans* appear to be regulated through sustained MPK‐1 activation (Lee et al., 2007). RSV maintained MPK‐1 activity during aging, whereas MPK‐1 activity dramatically decreased with age in EtOH‐treated worms (Figure 2a,b). Thus, we suggest that sustained MPK‐1 activation might be an important factor in the RSV‐mediated maintenance of germline mitotic cells during aging. These results suggest that sustained MPK‐1 activation might be an important factor in RSV‐mediated maintenance of GSCs and progenitor cells during aging. It is known that *sir‐2.1(ok434)* mutant worms are short‐lived and stress‐sensitive, while overexpression of *sir‐2.1* induces lifespan extension in a *daf‐16‐*dependent manner (Tissenbaum & Guarente, 2001; Wang & Tissenbaum, 2006). Fertility can be reduced in short‐lived worms (Gems & Riddle, 2000). Although loss of germ cells extends *C. elegans* lifespan through regulation of DAF‐16 (Berman & Kenyon, 2006), the lifespan extension induced by certain mutations occasionally requires the presence of an intact adult germline and the continuous production of mature eggs (Greer et al., 2010). Thus, the relationship between lifespan and reproduction can be more complex than we might think. In this study, we observed that adult lifespan of *sir‐2.1(ok434)* mutant worms was shorter than that of WT worms. Nevertheless, the *sir‐2.1(ok434)* mutant worms had more class III germlines compared to the WT worms on day 10 of adulthood (Figure 6). In addition, Figure 7 shows that *sir‐2.1(ok434)* mutant worms had increased brood size (280.6 viable progeny)) compared to WT worms (259.1 viable progeny). It has been recently reported that initiation of germline apoptosis promotes gonad senescence in *C. elegans *(de la Guardia et al., 2016). SIR‐2.1 translocates from the nucleus into the cytoplasm and the translocation event is related to DNA damage‐induced apoptosis, known as an early event in germ cell apoptosis (Greiss, Hall, Ahmed, & Gartner, 2008). Taken together, loss of function of SIR‐2.1 may protect the *C. elegans* germline against DNA damage‐induced apoptosis as *sir‐2.1(ok434)* mutant worms seem to have improved germline status compared to WT worms. Although such an idea seems likely, much of this system remains unclear. Thus, further studies are required to clarify the genetic pathways between *sir‐2.1* and germline aging during *C. elegans* aging process.

In summary, we novelly identified MPK‐1/ERK as a potent, critical RSV‐inducible factor, especially in terms of organismal longevity (Supporting Information Figure S5a) and GSC maintenance (Supporting Information Figure S5b). Given that MPK‐1 and SKN‐1 are highly conserved from *C. elegans* to mammals, these findings have important implications in utilizing RSV to improve the outcome of diseases associated with aging, diabetes, and cancer in mammals, including humans.

## 4 EXPERIMENTAL PROCEDURES

### 4.1 *C. elegans* strains

All *C. elegans* strains were maintained at 20°C as described previously (Brenner, 1974). We used the wild‐type Bristol strain N2 as well as the mutants and transgenic worms are listed in Supporting Information Table S1. *sir‐2.1(ok434)* mutants were outcrossed four times to wild‐type (N2) worms (Supporting Information Figure S1). For more details, please see Supporting Information Table S1.

### 4.2 Resveratrol preparation and lifespan assay

RSV (Sigma, St. Louis, MO, USA) preparation and lifespan assays were performed as described in the Supporting Information Appendix S1.

### 4.3 Western blot

Western blot analysis was performed as described in the Supporting Information Appendix S1.

### 4.4 RNA interference

RNA interference experiments were performed as described in the Supporting Information Appendix S1.

### 4.5 Real‐time quantitative polymerase chain reaction

Real‐time quantitative polymerase chain reaction (qRT–PCR) experiments were performed as described in the Supporting Information Appendix S1.

### 4.6 Immunohistochemistry

Immunohistochemistry experiments were performed as described in the Supporting Information Appendix S1.

### 4.7 Reproductive span analysis

Reproductive span‐related experiments were performed as described in the Supporting Information Appendix S1.

### 4.8 Statistics

Statistical significance from the lifespan and reproductive span assays was analyzed by logrank (Mantel–Cox) and analysis of variance (ANOVA) tests. Data are presented as mean ± standard deviation. Statistical significance of the phenotypes of *C. elegans* germline and brood size was calculated using the two‐tailed Student’s *t* test, and the error bars indicate standard deviation (*SD*).

## CONFLICT OF INTEREST

The authors declare that they have no conflict of interests.

## **AUTHOR’S** **CONTRIBUTIONS**

DSY and M‐HL performed study conception and design. DSY, DSC, YC, JWL, and M‐HL carried out experiments. DSY, DSC, YC, JWL, and M‐HL conducted analysis and interpretation of data. DSY drafted the manuscript, and M‐HL reviewed the manuscript.
