# Supplementary material for: MPK‐1/ERK is required for the full activity of resveratrol in extended lifespan and reproduction
Source: Aging Cell. 2018 Dec 21;18(1):e12867. doi: 10.1111/acel.12867 (PMC6351825; doi:10.1111/acel.12867)
Supplement: Supplementary file 1 [file ACEL-18-e12867-s001.docx]

**MPK-1/ERK is required for the full activity of resveratrol in extended lifespan and reproduction**

Dong Suk Yoon^1,2^, Dong Seok Cha^3^, Yoorim Choi^2,4^, Jin Woo Lee^2,4,5^, Myon-Hee Lee^1,6^*

^1^Department of Medicine, Brody School of Medicine at East Carolina University, Greenville, North Carolina 27834, USA

^2^Department of Orthopaedic Surgery, Yonsei University College of Medicine, Seoul 03722, South Korea

^3^Department of Oriental Pharmacy, College of Pharmacy, Woosuk University, Jeonbuk 55338, South Korea

^4^Brain Korea 21 PLUS Project for Medical Science, Yonsei University College of Medicine, Seoul 03722, South Korea

^5^Severance Biomedical Science Institute, Yonsei University College of Medicine, 50-1 Yonsei -ro, Seodaemun-gu, Seoul 03722, South Korea

^6^Lineberger Comprehensive Cancer Center, University of North Carolina-Chapel Hill, Chapel Hill, North Carolina 27599, USA

**SUPPORTING INFORMATION**

**SUPPORTING INFORMATION LISTING**

**Supplemental Methods.**

**Supplemental Table 1.** A list of *C. elegans* strains used for this study.

**Supplemental Table 2.** A list of antibodies that were used for this study.

**Supplemental Table 3.** Statistical significance between EtOH and 100 µM RSV group was determined by the log-rank (Mantel-Cox) test. Changes in mean lifespan are shown as comparisons with EtOH-treated groups of each strain (%).

**Supplemental Table 4.** A list of primers for qRT-PCR that were used for this study

**Supplemental Figure S1.** Outcrossing (4×) of *sir-2.1*(*ok434*) mutant worms.

**Supplemental Figure S2.** RSV increases phosphorylation of MPK-1 protein.

**Supplemental Figure S3.** Both MPK-1 and SIR-2.1 are necessarily required for RSV-enhanced oxidative stress resistance.

**Supplemental Figure S4.** qRT-PCR and western blot analyses for DAF-16 and its targets by RSV treatment.

**Supplemental Figure S5.** Proposed models of RSV-mediated MPK-1 action.

**Supplemental Figure S6.** The depletion of *sir-2.1* expression by RNAi.

**SUPPORTING INFORMATION**

**Supplemental Methods:**

**Resveratrol preparation and lifespan assay**

RSV (Sigma, St. Louis, MO, USA) was dissolved in ethanol (EtOH) to stock concentrations of 25, 50, 100. and 200 mM. Agar plates for lifespan assay consist of nematode growth medium (NGM) with 0.1% EtOH or 100 μM RSV. RSV was directly added to the NGM solution before pouring the solution into petri dishes. To prevent possible developmental defects caused by RNA interference or RSV, all lifespan experiments were started using synchronized worms at 4 days after embryo stage. The worms were transferred to the EtOH- or RSV-containing NGM agar plates. All worms tested were transferred to fresh plates every 2 days and their survival rates were counted daily. Worms that showed no response to touch with a platinum wire and no pharyngeal pumping were considered as dead.

**RNA interference (RNAi)**

RNAi constructs were obtained from the *C. elegans* ORF RNAi Library (Thermo Fisher Scientific). RNAi experiments were performed by feeding bacteria expressing double stranded RNAs corresponding to *mpk-1*, *daf-16*, and *skn-1*, genes at 4 days after embryo stage. L4440 vector was used as a negative RNAi control. The results for RNAi experiments were pooled from at least three independent experiments. The RNAi plates for lifespan assays contained the 100 μM RSV or equal volume of EtOH (0.1%). There are no available RNAi bacteria corresponding to *sir-2.*1 in the ORF RNAi Library used in this study. To obtain bacteria expressing double stranded RNAs corresponding to *sir-2.1*, a RNA interference (RNAi) construct was generated by RT-PCR amplification of fragments by using gene-specific oligonucleotides and cloning directly into L4440 by using *HindIII* and *XhoI* restriction enzymes (see Supporting Information Figure S6). The *sir-2.1*::L4440 plasmids were transformed into a RNase III-deficient (*rnc^-^*) *E. coli* strain (HT115) ([Kamath *et al.* 2001](#_ENREF_1)), which is broadly used for feeding RNAi experiments in *C. elegans*.

**Immunohistochemistry**

Immunostaining was performed as described previously ([Yoon *et al.* 2016](#_ENREF_4)). *C. elegans* worms were dissected, fixed with 3% paraformaldehyde/0.1M K_2_HPO_4_ (pH 7.2), and then post-fixed in cold methanol. The fixed gonads were blocked with 0.5% bovine serum albumin solution dissolved in the 1× phosphate-buffered saline containing 0.1% tween 20 (1× PBST-BSA solution) for 30 minutes. After washing three times, the dissected gonads were incubated in primary antibodies diluted in 1× PBST-BSA solution for 2 hours at room temperature. After washing with 1× PBST-BSA solution three times, the dissected gonads were incubated with secondary antibodies for 2 hours at room temperature in the dark. The nuclei were stained with 4,6-diamidino-2-phenyindole (DAPI). See Table S2.

**Western blot**

For the preparation of whole protein samples, collected *C. elegans* worms were lysed in Laemmli sample buffer (Bio-Rad Laboratories, Inc., Hercules, CA, USA) by boiling for 10 minutes. The lysed proteins analyzed by 10% sodium-dodecyl sulfate-polyacrylamide gel electrophoresis (SDS-PAGE) (Bio-Rad Laboratories, Inc.). Transferred membranes were blocked with 5% nonfat dry milk (Biotium, Inc., Fremont, CA, USA) in tris-buffered saline (TBS) with 0.5% Tween-20 (TBST) and primary antibody incubations were performed in 1% (w/v) nonfat dry milk in TBST for 12 hours with antibodies at 4°C. Secondary antibodies were incubated in 5% (w/v) nonfat dry milk in TBST for 1 hour at room temperature. Western blot images were obtained using the LI-COR C-DiGit Chemiluminescence Western Blot Scanner and Image software (LI-COR, Lincoln, Nebraska, USA). Please see Table S2 for the list of antibodies used for this study.

**Measurement of reactive oxygen species using 2’,7’-dichlorofluorescein diacetate (DCFDA)**

Intracellular ROS levels were measured using DCFDA (Molecular Probes, Carlsbad, CA, USA) as described previously ([Yoon *et al.* 2017](#_ENREF_2)). Briefly, synchronized L1 stage worms were placed onto EtOH- or RSV-containing RNAi plates, and then the worms were maintained for 2 days at 20°C. For DCFDA assay, approximately 100 worms at L2−L3 larvae stage (day 2 from embryos) per a well were transferred into the 96-well plate containing 50 μL of M9 buffer. Immediately, 50 μL of 50 μM H2DCF-DA was added to each well. The basal fluorescence was quantified using a microplate fluorescence reader at an excitation wavelength of 492 nm and an emission wavelength of 527 nm. Fluorescence was measured every hour. Three replicate experiments were conducted.

**Real-time quantitative polymerase chain reaction (qRT-PCR)**

Total RNA was isolated using an RNeasy kit (Qiagen, Hilden, Germany) according to the manufacturer’s instruction. Total RNA (1 µg) was then reverse-transcribed using an Omniscript® kit (Qiagen). Quantitative real-time polymerase chain reaction was performed as previously described ([Yoon *et al.* 2014](#_ENREF_3)). Primers used in this study are listed in Supplemental Table 4. Mean cycle threshold values from duplicate samples obtained in three independent experiments were used to calculate mRNA expression, with normalization to *acts* as an internal control.

**Reproductive span analysis**

All experiments were conducted at 20°C. Synchronized L1 larvae were plated on NGM plates seeded with OP50 *E. coli* and allowed to grow to the L4 larvae stage. When the worms reached the L4 larvae stage, 20 worms per group were picked individually to new NGM plates with OP50 *E. coli*. The next day, worms capable of producing progeny were transferred to EtOH- or RSV-containing plates in which each RNAi bacteria was seeded. Worms that died during the reproductive periods were excluded in this experiment. The worms were transferred daily to new plates with same condition and viable progenies on the old plates were counted daily two days after the worms were moved to the new plate. The last day where viable progeny were produced was determined as the day of reproductive cessation for each individual. For analyses of brood size, reproductive span, and number of progeny, all experiments were performed with 10 worms per group in triplicate.

**Supplemental Table 1. A list of *C. elegans* strains used for this study**

| **Strain** | **Genotype** | **Source** |
| --- | --- | --- |
| N2 | wild-type | CGC |
| VC199 | *sir-2.1(ok434) IV* | CGC |
| CF1038 | *daf-16(mu86) I* | CGC |
| QV225 | *skn-1(zj15) IV* | CGC |
| HE1006 | *rol-6(su1006) II* | CGC |
| GA468 | *geIs3 [sir-2.1(+) + rol-6(su1006)]* | CGC |
| LD1 | *Is007 [skn-1b/c::GFP + rol-6(su1006)]* | Blackwell lab. |
| CF1139 | *muIs61 [(pKL78) daf16::GFP + rol-6(su1006)]* | CGC |
| SD939 | *mpk-1(ga111) unc-79(e1068) III*, temperature-sensitive sterile | CGC |

**Supplemental Table 2. A list of antibodies that were used for this study**

| **Antibodies** | **Target proteins** | **Dilution** | **Sources** |
| --- | --- | --- | --- |
| ERK-1 | MPK-1 | 1:1000 | Santa Cruz (Cat#: (K-23): sc-94) |
| MAPK(YT) | Active MPK-1 | 1:1000 | Sigma (Cat#: M8159) |
| SIR-2.1 | SIR-2.1 | 1:1000 | Thermo Scientific (Cat#: PA1-16933) |
| α-TUBULIN | α-TUBULIN | 1:1000 | Sigma (Cat#: T6074) |
| HIM-3 | HIM-3 | 1:500 | Novus (Cat#: 53470002SS) |
| GFP | GFP | 1:5000 | Abcam (Cat#: ab6556) |
| mAb414 | Nuclear Pore Proteins | 1:500 | Abcam (Cat#: ab24609) |

**Supplemental Table 3. Statistical significance between EtOH and 100 µM RSV group was determined by the log-rank (Mantel-Cox) test. Changes in mean adult lifespan in shown as compared with EtOH groups of each strain (%)**

| **Strain** | **Mean Adult Lifespan (days)** | | **Changes in Adult Lifespan (%)** | **Log-Rank Test** | **Figures** |
| --- | --- | --- | --- | --- | --- |
|  | **EtOH**  **(n)** | **RSV**  **(n)** |  |  |  |
| wild-type (N2); control RNAi | 17.429±0.300  (156) | 20.919 ± 0.276  (160) | 20.284  ± 6.806 | *p* < 0.001 | 1b |
| *sir-2.1(ok434)*; control RNAi | 14.953±0.314  (151) | 16.384 ± 0.257  (138) | 9.994  ± 4.102 | *p* = 0.038 | 1c |
| wild-type (N2); *mpk-1* RNAi | 14.465±0.192  (157) | 15.418 ± 0.181  (170) | 6.738  ± 0.971 | *p* < 0.001 | 1e |
| *sir-2.1(ok434)*; *mpk-1* RNAi | 13.669±0.265  (139) | 13.730 ± 0.247  (141) | 0.535  ± 2.754 | *p* = 0.058 | 1f |
| wild-type (N2); control RNAi | 17.183±0.253  (142) | 20.466 ± 0.295  (131) | 19.014  ± 0.756 | *p* < 0.001 | 3a |
| wild-type (N2); *skn-1* RNAi | 15.533±0.240  (152) | 16.777 ± 0.243  (148) | 8.205  ± 3.413 | *p* < 0.001 | 3b |
| wild-type (N2); *daf-16* RNAi | 14.769±0.230  (156) | 16.023 ± 0.181  (171) | 8.420  ± 0.352 | *p* = 0.001 | 3c |
| *sir-2.1(ok434)*; control RNAi | 15.299±0.311  (147) | 17.014 ± 0.293  (140) | 11.201  ± 0.395 | *p* = 0.001 | 3e |
| *sir-2.1(ok434)*; *skn-1* RNAi | 14.460±0.280  (139) | 14.608 ± 0.238  (148) | 1.022  ± 1.621 | *p* = 0.597 | 3f |
| *sir-2.1(ok434)*; *daf-16* RNAi | 13.138±0.228  (138) | 14.536 ± 0.237  (138) | 10.615  ± 2.601 | *p* < 0.001 | 3g |
| *daf-16(mu86)*; control RNAi | 13.967±0.251  (183) | 15.335 ± 0.213  (194) | 10.209  ± 1.448 | *p* < 0.001 | 4a |
| *daf-16(mu86)*; *sir-2.1* RNAi | 14.000±0.216  (198) | 15.269 ± 0.221  (186) | 9.030  ± 0.721 | *p* < 0.001 | 4b |
| *daf-16(mu86)*; *mpk-1* RNAi | 12.960±0.194  (197) | 12.962 ± 0.190  (213) | 0.242  ± 0.441 | *p* = 0.851 | 4c |
| *daf-16(mu86)*; *skn-1* RNAi | 13.676±0.218  (185) | 13.681 ± 0.233  (188) | 0.029  ± 0.424 | *p* = 0.583 | 4d |
| *skn-1(zj15)*; control RNAi | 12.835±0.199  (200) | 14.168 ± 0.195  (208) | 10.366  ± 1.046 | *p* < 0.001 | 4g |
| *skn-1(zj15)*;  *mpk-1* RNAi | 12.834±0.178  (193) | 14.005 ± 0.182  (202) | 9.330  ± 0.927 | *p* < 0.001 | 4h |
| *skn-1(zj15)*;  *sir-2.1* RNAi | 12.981±0.196  (211) | 13.086 ± 0.194  (221) | 0.812  ± 0.736 | *p* = 0.611 | 4i |
| *skn-1(zj15)*; *daf-16* RNAi | 12.276±0.162  (221) | 12.389 ± 0.173  (221) | 1.024  ± 0.682 | *p* = 0.334 | 4j |

**Supplemental Table 4. A list of primers for qRT-PCR that were used for this study**

| **Targets** | **Sense** | **Anti-sense** |
| --- | --- | --- |
| *acts* | CTCTTGCCCCATCAACCATG | CTTGCTTGGAGATCCACATC |
| *daf-16* | ATGCGAATTCAGAATGAAGGA | CTTTGTAGTCGTCTCAATAGT |
| *skn-1* | TTCGGAGATGTCATTAAGCG | TGCGTCTTTGACGGCAAGTGCG |
| *dod-17* | CAGGAAATCTTATTCGGACTACTC | GTTAGCGACAGTGAGTGTG |
| *mdh-1* | CTCGTGACGATCTCTTCAACAC | GTCATAGACACCAGCCTTCTTGAG |
| *gpd-2* | CTCCATCGACTACATGGTCTACTTG | AGCTGGGTCTCTTGAGTTGTAGAC |
| *sod-3* | CGAGCTCGAACCTGTAATCAGCCATG | GGGGTACCGCTGATATTCTTCCAGTTG |
| *hsp-16.2* | CTGCAGAATCTCTCCATCTGAGTC | AGATTCGAAGCAACTGCACC |
| *ctl-1* | CGGATACCGTACTCGTGATGAT | CCAAACAGCCACCCAAATCA |

**Supporting Figure S1.** **Outcrossing (4×) of *sir-2.1*(*ok434*) mutant worms.** (a) Schematic of *sir-2.1* genomic DNA. Box, exon; connecting line, intron; ATG, initiation codon; TGA, termination codon; thin bar, deletion region; arrowheads, primer pairs used for genomic PCR. (b) Primer sequences. (c) PCR amplification of the targeted region (see Figure S1A) in the wild-type (*N2*) and *sir-2.1* (*ok434*) deletion mutants (crossing times: 0**×**, worms from CGC; 4**×**, outcrossing four times to wild-type). (d) The SIR-2.1 protein levels in the out-crossed *sir-2.1* mutants were confirmed by western blot analysis.

**Supporting Figure S2. RSV increases phosphorylation of MPK-1 protein.** Protein levels of SIR-2.1, total MPK-1, and phosphorylated MPK-1 (pMPK-1) were analyzed by western blot analysis. The protein level of α-tubulin was used as a loading control and the protein lysates from *mpk-1* (RNAi) and *sir-2.1* overexpressing worms (GA468) were used to confirm the size of MPK-1 and SIR-2.1 proteins. The worms tested were incubated in K-medium in the presence of EtOH or RSV (25, 50, 100, and 200 μM) for 6 hr. *C. elegans* has two MPK-1 isoforms, MPK-1A and MPK-1B. Band intensities were measured using ImageJ software and were normalized using α-tubulin to control for protein levels.

**Supporting Figure S3. Both MPK-1 and SIR-2.1 are necessarily required for RSV-enhanced oxidative stress resistance.** The effects of RSV on the time-course accumulation of intracellular ROS levels were determined in wild-type (N2) and *sir-2.*1(*ok434*) worms fed with control RNAi (a and b) or *mpk-1* RNAi (c and d) bacteria using 2, 7-dichlorofluorescein diacetate (DCF-DA; Molecular Probes). Animals (500) from each group were analyzed in triplicate, and the results are expressed as means ± S.D. of relative fluorescence units (RFU). **P* *<* 0.05; ***P* < 0.01; ****P* < 0.001. The levels of DCF fluorescence in all experiment groups were normalized to those of the basal levels measured at the starting time point.

**Supporting Figure S4. qRT-PCR and western blot analyses for DAF-16 and its targets by RSV treatment.** The mRNA and protein expression levels of *daf-16* was determined in wild-type (N2) and DAF-16::GFP (*muls61*) worms treated with EtOH or 100 μM RSV by qRT-PCR and western blot assays in triplicate. Wild-type and DAF-16::GFP worms synchronized at L1 stage were grow on OP50 bacteria-seeded NGM plates until they became adults (at 4 days after embryo stage), and then the worms were transferred to RNAi plates containing EtOH or 100 μM RSV. The worms were fed with empty vector, *sir-2.1*, or *mpk-1* vector-containing RNAi bacteria from day 1 to day 10 of adulthood. Likewise, EtOH or RSV was supplemented to the worms from day 1 to day 10 of adulthood. The worms on day 10 of adulthood were used for qRT-PCR (a) and western blot analyses (b). *ns* means no significance. (c) Quantitative analysis of DAF-16 protein was performed by ImageJ software in triplicate. * *P* < 0.05. (d) The mRNA levels of *sod-3* and *hsp-16.2*, known to be downstream targets of DAF-16 were determined in the wild-type worms by qRT-PCR. Likewise, the wild-type worms were fed with empty vector, *sir-2.1*, or *mpk-1* vector-containing RNAi bacteria and EtOH or RSV was supplemented to the worms from day 1 to day 10 of adulthood. * *P* < 0.05, ns; not significant compared to control.

**Supporting Figure S5. Proposed models on RSV-mediated MPK-1 action.** (a) RSV can activate SIR-2.1/sirtuin and phosphorylate MPK-1/ERK protein to extend the lifespan of *C. elegans*. RSV-mediated MPK-1 exerts its ability independently of SIR-2.1 by targeting a different downstream gene. Our model suggests that the MPK-1/SKN-1 pathway is a novel target of RSV and that this pathway operates in a SIR-2.1/DAF-16-independent manner under RSV stimulation. (b) The current study revealed a novel effect of RSV in maintaining mitotic germline stem cells (GSCs) of *C. elegans* throughout the lifespan of the worms. Although the positive effect of RSV on GSCs is not dependent on SIR-2.1, without MPK-1, RSV entirely loses its capacity to maintain healthy GSCs during aging. Thus, RSV-mediated MPK-1/ERK may represent a promising therapeutic target in small molecule-induced longevity and stem cell maintenance.

**Supporting Figure S6. The depletion of *sir-2.1* expression by RNAi.** (a) A Protein levels of SIR-2.1 were analyzed by western blot analysis. The protein level of α-tubulin was used as a loading control and the protein lysates from wild-type (N2), *sir-2.1*(*ok434*) mutant, *rol-6*(*su1006*), and *sir-2.1* overexpressing worms (*GA468*) were used for the western blot. (b) Schematic of *sir-2.1* genomic DNA. Box, exon; connecting line, intron; ATG, initiation codon; TGA, termination codon. Below bar, RNAi target region. (c) Western blot. *sir-2.1* (#1) and *sir-2.1* (#2) are different RNAi bacterial colonies.

**Supplemental Figure S1**


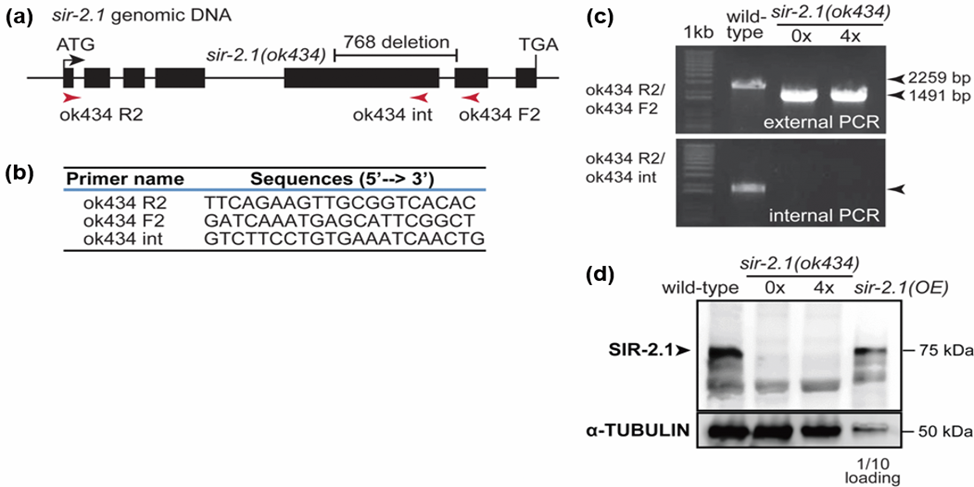


**Supplemental Figure S2**

**
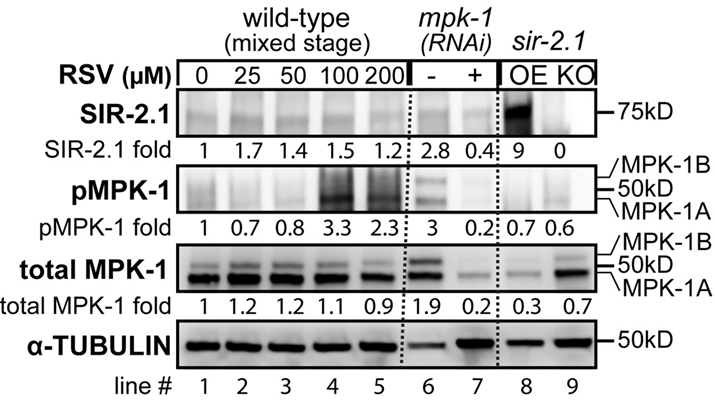
**

**Supplemental Figure S3**

**
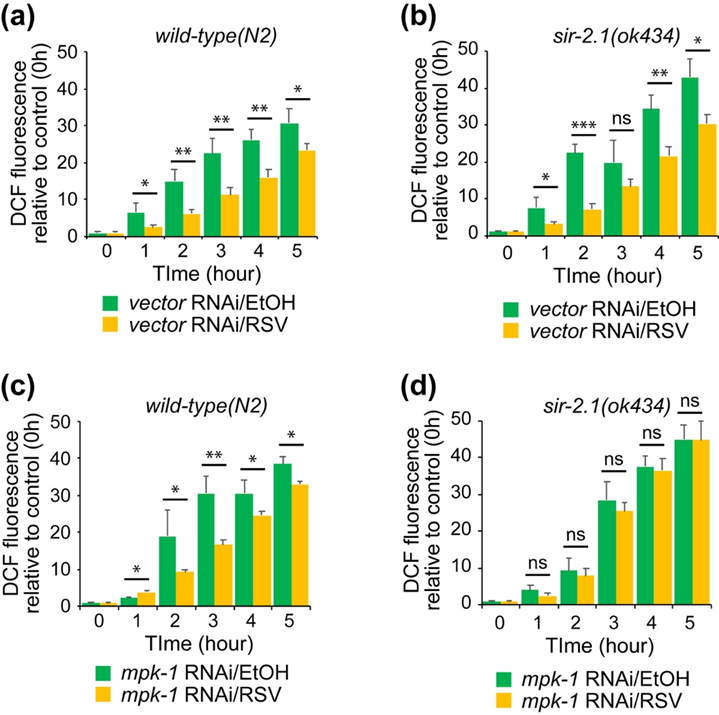
**

**Supplemental Figure S4**

**
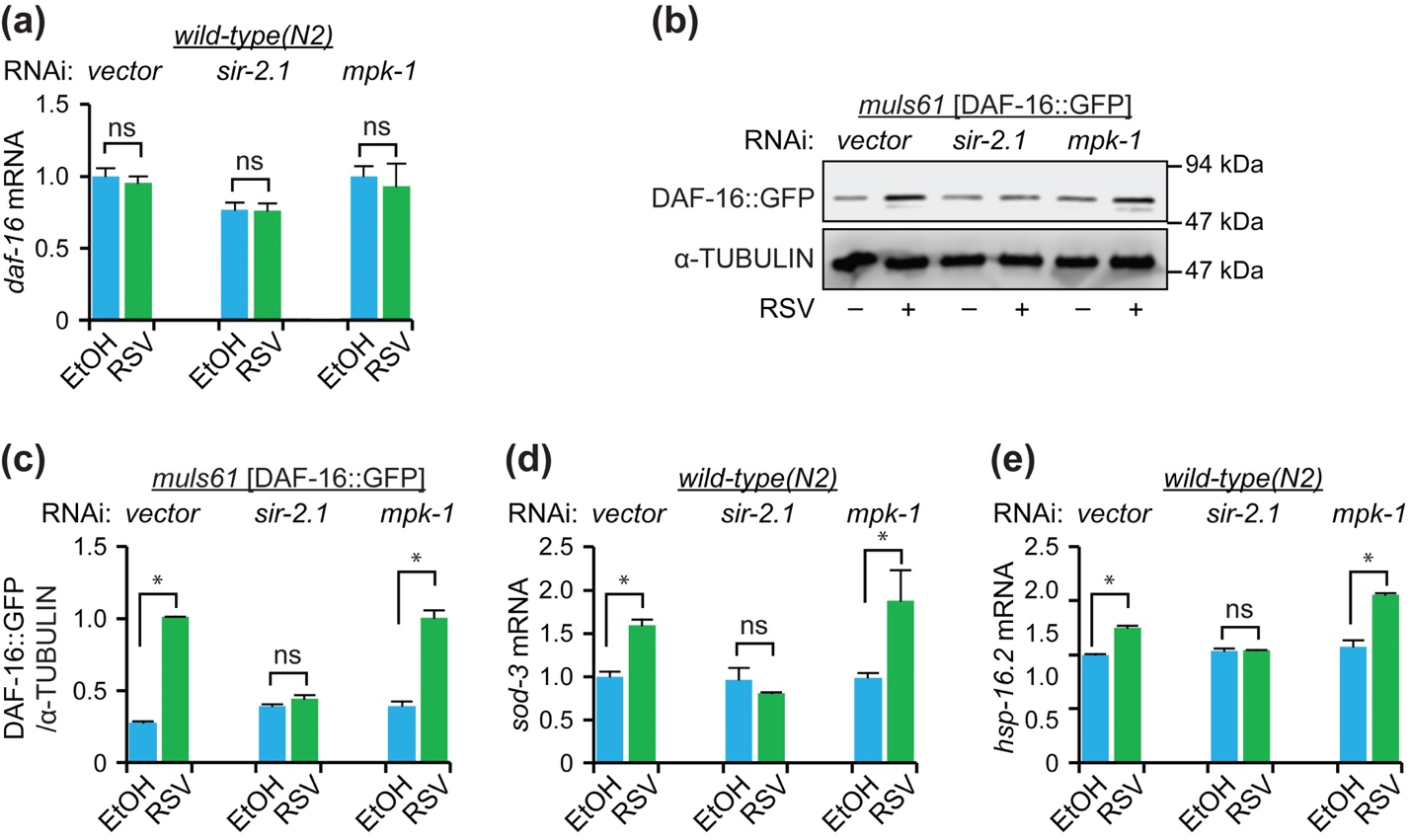
**

**Supplemental Figure S5**

**
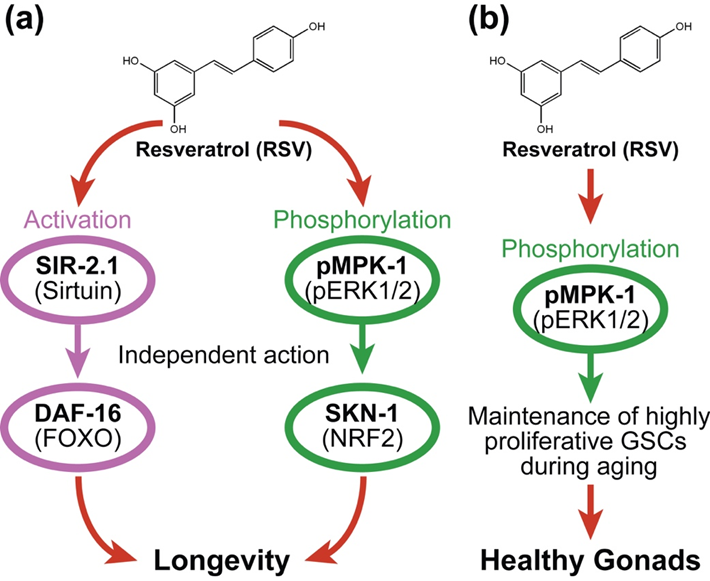
**

**Supplemental Figure S6**

**
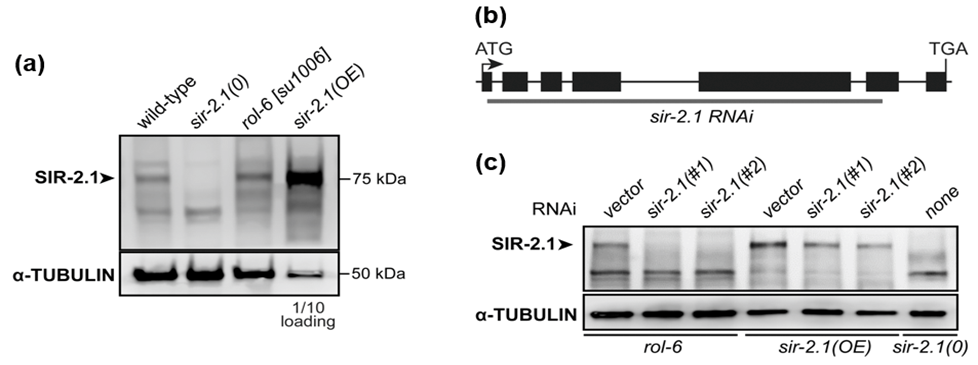
**

**REFERENCES FOR SUPPORTING INFORMATION**

Kamath, R.S., Martinez-Campos, M., Zipperlen, P., Fraser, A.G., & Ahringer, J. (2001). Effectiveness of specific RNA-mediated interference through ingested double-stranded RNA in Caenorhabditis elegans. *Genome Biology*, *2*, RESEARCH0002.

Yoon, D.S., Choi, Y., Cha, D.S., Zhang, P., Choi, S.M., Alfhili, M.A.,…. Lee, M.H. (2017). Triclosan Disrupts SKN-1/Nrf2-Mediated Oxidative Stress Response in C. elegans and Human Mesenchymal Stem Cells. *Scientific Reports,* *7*, 12592.

Yoon, D.S., Choi, Y., Jang, Y., Lee, M., Choi, W.J., Kim, S.H., Lee, J.W. (2014). SIRT1 directly regulates SOX2 to maintain self-renewal and multipotency in bone marrow-derived mesenchymal stem cells. *Stem Cells,* *32*, 3219-3231.

Yoon, D.S., Pendergrass, D.L., Lee, M.H. (2016). A simple and rapid method for combining fluorescent in situ RNA hybridization (FISH) and immunofluorescence in the C. elegans germline. MethodsX, *3*, 378-385

Kamath RS, Martinez-Campos M, Zipperlen P, Fraser AG, Ahringer J (2001). Effectiveness of specific RNA-mediated interference through ingested double-stranded RNA in Caenorhabditis elegans. *Genome biology*. 2, RESEARCH0002.

Yoon DS, Choi Y, Cha DS, Zhang P, Choi SM, Alfhili MA, Polli JR, Pendergrass D, Taki FA, Kapalavavi B, Pan X, Zhang B, Blackwell TK, Lee JW, Lee MH (2017). Triclosan Disrupts SKN-1/Nrf2-Mediated Oxidative Stress Response in C. elegans and Human Mesenchymal Stem Cells. *Scientific reports*. 7, 12592.

Yoon DS, Choi Y, Jang Y, Lee M, Choi WJ, Kim SH, Lee JW (2014). SIRT1 directly regulates SOX2 to maintain self-renewal and multipotency in bone marrow-derived mesenchymal stem cells. *Stem Cells*. 32, 3219-3231.

Yoon DS, Pendergrass DL, Lee MH (2016). A simple and rapid method for combining fluorescent in situ RNA hybridization (FISH) and immunofluorescence in the C. elegans germline. *MethodsX*. 3, 378-385.
